# New perspectives under heterogeneity: mechanism of nutrient heterogeneity on Cd-induced hormesis of soil respiration

**DOI:** 10.3389/fmicb.2025.1647658

**Published:** 2025-08-12

**Authors:** Qian Gao, Jiangshan Yang, Junyang Wu, Yongli Zhu, Diwu Fan, Jiangang Han

**Affiliations:** ^1^Co-Innovation Center for the Sustainable Forestry in Southern China, College of Ecology and Environment, Nanjing Forestry University, Nanjing, China; ^2^National Positioning Observation Station of Hung-tse Lake Wetland Ecosystem in Jiangsu Province, Hongze, China; ^3^School of Chemical Engineering and Materials, Changzhou Institute of Technology, Changzhou, China

**Keywords:** hormesis, cadmium, spatial heterogeneity of soil nutrients, soil respiration, bacterial community composition

## Abstract

Hormesis is a phenomenon in which low doses of toxins stimulate organisms, while high doses have inhibitory effects. Soil heterogeneity nutrient spatial profoundly influences community structure and stress responses by altering the microenvironment around microorganisms. Studies on hormesis in soil ecosystems have made significant progress, but most of them have overlooked the impact of soil nutrient spatial heterogeneity on hormesis. To examine the effects of nutrient spatial heterogeneity on the hormesis of soil ecosystem under cadmium (Cd) stress, we constructed three patches with different spatial nutrient distribution but the same total nutrient content through different spatial discharge patterns. Different levels of Cd stress were applied to the patches, and after incubation, soil respiration rate, content of dissolved organic matter (DOM) and metagenomic characteristics were measured. The results indicated that higher nutrient spatial heterogeneity enhanced the tolerance of soil respiration to Cd stress (the maximum stimulating dose increased from 0.03 to 3.0 mg·kg^−1^), and simultaneously improved the compensation capacity (*Hor*_*zone*_ increased from 0.04 to 21.59). The results also revealed that Cd stress had the least impact on soil microbial diversity of the high heterogeneity samples. The content of DOM initially displayed a hormesis-like phenomenon with increasing Cd stress, followed by a linear increase. Notably, the biphasic change trend became more pronounced as the degree of spatial heterogeneity increased (The maximum stimulation rate of DOM content increases from 10.8 to 22.9%). The hormetic response of soil respiration to nutrient spatial heterogeneity offers novel insights for the identification and mitigation of Cd pollution in ecosystems.

## Graphical Abstract

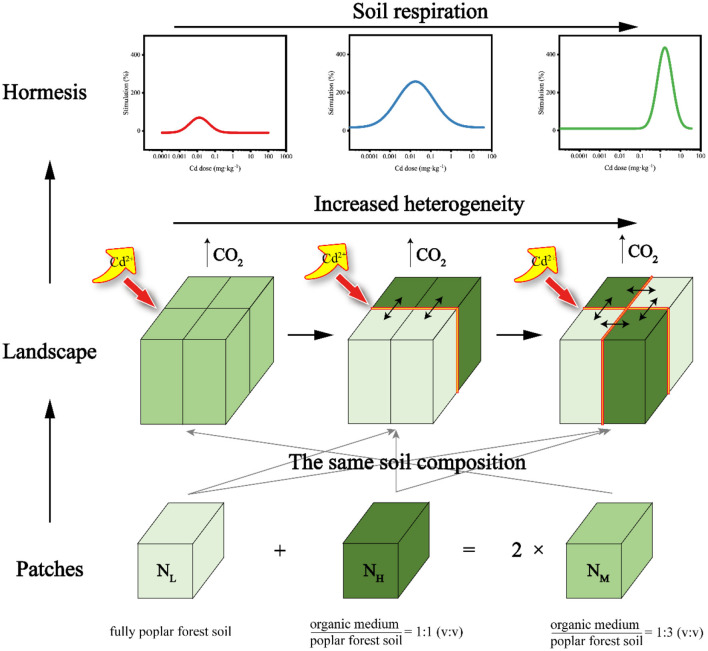

## 1 Introduction

Hormesis refers to the phenomenon that organisms produce stimulus response under low dose stimulation, but show inhibition under high dose stress. Calabrese believe that hormesis is an overcompensation mechanism, which responds to the system's stability before it collapses (Calabrese and Baldwin, 2002). In the classical framework of hormesis research, plants, animals, and microorganisms are used as experimental models, and corresponding biomass, growth rate, and immunity are set as the test endpoints (Agathokleous and Calabrese, 2019; Sebastiano et al., 2022; Agathokleous et al., 2021). In recent years, some studies have begun to use complex ecosystems as new experimental subjects. This approach helps reveal the mechanisms of Hormesis at the ecosystem scale and lays a foundation for further exploring the path of Hormesis at various scales, including the individual, community, and ecosystem levels (Fan et al., 2016; Yin et al., 2016; Wang et al., 2021). Among all the endpoints, soil respiration possesses distinctive advantages in terms of sensitivity and maintaining soil integrity, allowing it to effectively reflect the differences of soil samples with diverse spatial structures after being stressed by heavy metals (Alfaro et al., 2021; Chen et al., 2014; Choi et al., 2017).

Natural landscapes (the ecosystems with distinct nutrient heterogeneity and spatial pattern, which is distinguished from “artificial landscapes”) often exhibit high heterogeneity, including spatial patterns of sources (spatial heterogeneity of water and nutrients) and species (species diversity and spatial distribution; Fritsch et al., 2011; Xiao, 2021; Curd et al., 2018). One important form of nutrient heterogeneity is the coexistence of patches with different nutrient contents in the landscape. Under the influence of environmental dependence of microbial communities or inter-species relationships, microorganisms may exhibit different competitive or cooperative relationships in different nutrient patches (Bahram et al., 2018; Delgado-Baquerizo et al., 2020, 2016). For adjacent patches with different nutrient contents, nutrient diffusion at the patch boundaries forms non-patch-responsive areas (i.e., nutrient diffusion corridors). The heterogeneous diffusion speed of soil nutrients leads to dynamic variations in the distribution of soil dissolved organic matter (DOM) within this region. This phenomenon results in the expansion of boundary zones, consequently inducing distinct microbial activities in these corridor areas compared to adjacent patches (Fenchel, 2002; Fierer and Lennon, 2011; Martiny et al., 2006). Therefore, the inter-species relationships of soil microorganisms in nutrient heterogeneous landscapes are significantly different from those in homogeneous landscapes. When the landscape as a whole is threatened, this difference in microbial inter-species relationships can also lead to differences in responses to the threat, ultimately manifesting as changes in the function of the landscape as a whole (Van Den Hoogen et al., 2019; Tedersoo et al., 2012). Although there has been considerable progress in the study of ecosystem Hormesis, these studies have all been conducted on homogeneous systems. For heterogeneous landscapes, the Hormesis characteristics of these systems are still largely unexplored.

It is assumed that the changes in the relationship between microbial populations and communities and their impact on DOM stability can be reflected by changes in soil respiration. In this study, a landscape composed of three patches with different nutrient contents, including a homogeneous landscape and a landscape composed of two different nutrient content patches, was simulated. Using Cd as a stress agent, the soil respiration of the entire landscape as the endpoint, this study investigates the response differences of the three landscapes under different stress doses, and further analyzes the changes in microbial community structure and diversity, as well as the differences in DOM content. Totally, this provides a basis for assessing and regulating heavy metal pollution effects at the landscape scale.

## 2 Materials and methods

### 2.1 Experiment design

**Figure 1:**
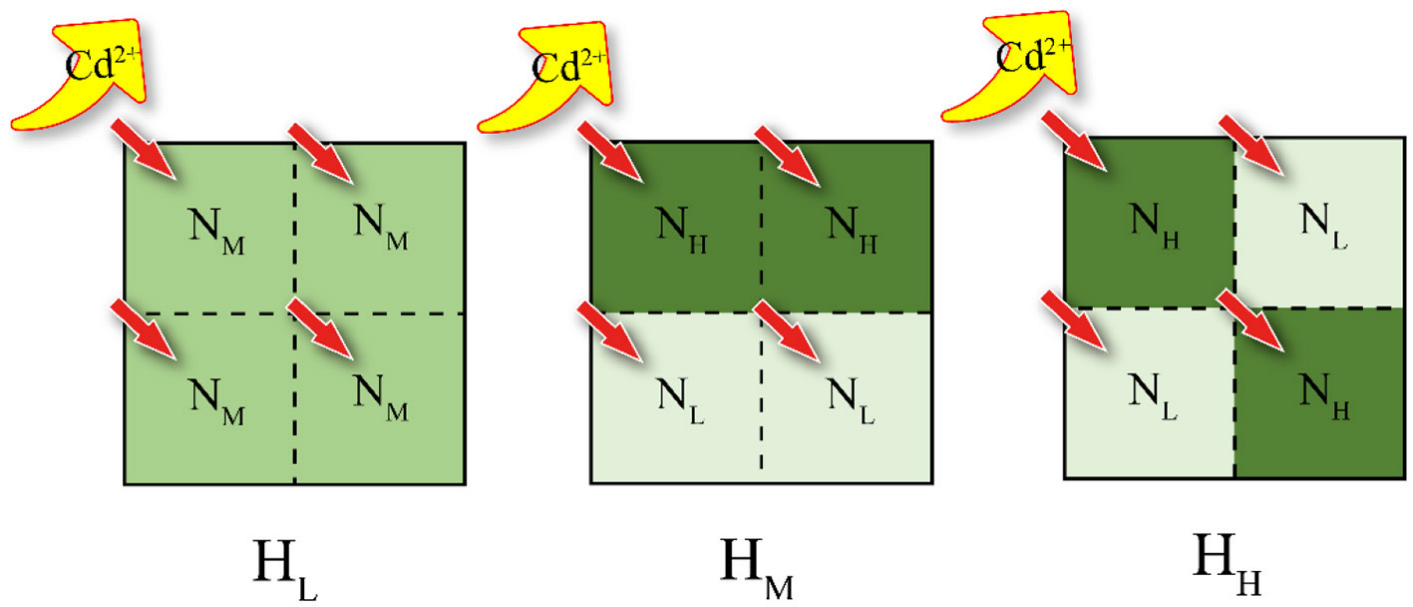
Experimental design schematic. The experiment includes three soil configuration treatments, which are homogeneous soil (H_L_), large patch heterogeneous soil (H_M_), and small patch heterogeneous soil (H_H_). These three types of soil compositions differ in nutrient content, with N_L_, N_M_, and N_H_ representing low, medium, and high nutrient levels, respectively. Furthermore, the proportion of original soil to nutrient-rich soil in N_M_ is equal to the sum of N_L_ and N_H_.

In this study, the collected wetland agricultural soil (from 0 to 20 cm depth, Hung-tse Lake Wetland, Jiangsu, China) and the purchased organic medium (purchased from Jiangsu Xingnong Fertilizer Co., LTD.) were used to conduct a simulating lab experiment. The chemical properties of the air-dried poplar forest soil and cultivation soil are presented in Supplementary Table S1. The patches were divided into N_L_ (fully air-dried poplar forest soil), N_M_ [organic medium: air-dried poplar forest soil = 1:3 (v: v)], and N_H_ [organic medium: air-dried poplar forest soil = 1:1 (v: v)] according to the amount of organic matrix content (The specific qualities are shown in Supplementary Table S2). These patches were arranged in various combinations to create three heterogeneous treatments (Figure 1). Each treatment was replicated four times, resulting in a total of 12 pots.

Each pot (11 cm × 11 cm × 10 cm) was divided into four equal-sized patches. Prior to filling the pots with soil, a sterile sealing film was placed at the bottom of each pot to prevent water loss through the drainage holes. There were no physical barriers present among patches, ensuring unrestricted permeation and liquid flow. According to the degree of soil heterogeneity, it was divided into three levels, H_L_, H_M_ and H_H_. The H_L_ treatment consisted of four N_M_ patches, and both H_M_ and H_H_ treatments consisted of two N_H_ and two N_L_ patches. This was done to ensure the same amount of total organic matrix within each treatment. The spatial distribution of patches in the different treatments is shown in Figure 1.

Prior to the experiment, soil samples from each patch type were thoroughly mixed and stored in light-protected conditions for over 30 days. At the start of pre-cultivation, ultrapure water (30%) was added to activate soil microorganisms, and pre-cultivated at a humidity of 30% and temperature of 30°C for 48 h. After pre-cultivation, individual patches were combined into a heterogeneous design with homogeneous soil to avoid bias. At the beginning of the experiment, 50 ml of CdCl_2_ solution (with concentrations of 0.03, 0.06, 0.3, 0.6, 3.0, 6.0, and 30.0 mg·kg^−1^, and 0 for adding 50 ml of ultrapure water) was evenly sprayed on the surface of each sample (regardless of the type of patch). Then, samples were cultivated under the same conditions as the pre-cultivation for 24 h.

### 2.2 Measurements

#### 2.2.1 Soil respiration rate

After the Cd culture was completed, the soil sample was sealed in a closed acrylic container. Twenty milliliter of headspace gas was extracted from the bottle with a syringe (Supplementary Figure S5). An air sample was taken every 20 min for a total of 4 times as parallel samples (0 min as the control). The air samples were injected into the gas chromatograph at a constant and slow speed. High-purity nitrogen and helium, as well as a mixture of 95% argon and 5% methane, were used as carrier gases. The standard gas was provided by the National Institute of Metrology, China (No. 595669), and high-purity nitrogen was used as the purge gas. The CO_2_ concentration in the sample was calculated based on the peak area ratio between the standard gas and the test gas.

The CO_2_ concentration was analyzed by a gas chromatograph (7890A, Agilent Technologies, USA).

#### 2.2.2 Dissolved organic carbon

After completing the soil respiration test, the soil samples are subjected to freeze-drying for 48 h at a temperature of −80°C and a pressure below 1 Pa. Subsequently, 10.0 g of dried soil sample is weighed, followed by addition of 50 ml of ultrapure water. The mixture is then vigorously shaken and centrifuged, after which the supernatant is collected through a 0.45 μm filter membrane. The dissolved organic carbon (DOC) content and 3D fluorescence spectra were measured simultaneously.

The concentrations of DOC and the corresponding dissolved organic matter (DOM) were analyzed using total organic carbon analyzer (Multi N/C 3100, Analytik Jena, Germany). The 3D fluorescence spectra were analyzed using fluorescence spectrophotometer (LS-55, PerkinElmer, USA).

#### 2.2.3 Soil microbial community

The microbial samples were obtained from freeze-dried and well-mixed soil samples (no separate sampling was conducted for the boundary areas). A homogenized dry soil sample was collected to extract total DNA from microbial community samples of diverse origins using the CTAB method. The quality of the DNA extraction was assessed through agarose gel electrophoresis, and its quantity was measured using NanoDrop (Thermo Fisher Scientific Inc., Massachusetts, USA) a UV spectrophotometer. PCR amplification targeting the V3–V4 region was performed with primers 341F 5′-CCTACGGGNGGCWGCAG3′) and 805R 5′-GACTACHVGGGTATCTAATCC3′), which were subsequently confirmed by agarose gel electrophoresis. Purification of the PCR products involved AMPure XT beads (Beckman Coulter Genomics, Danvers, MA, USA), followed by quantification using Qubit (Invitrogen, USA). Evaluation of purified PCR products included an Agilent 2100 Bioanalyzer system (Agilent Technologies Inc., Santa Clara CA) in conjunction with Illumina's library quantification kit from KapaBiosciences Inc., Woburn MA; libraries were considered acceptable if their concentration exceeded 2 nmol·L^−1^.

### 2.3 Calculation of the stimulation rate and fitting of the dose-response relationship

The dose-response curves were generated by employing the CO_2_ emission rate, relative abundance of bacteria, and community diversity as variables. The calculation method for determining stimulation rates (sti, %) is outlined as follows:


$$\begin{array}{l} R=\frac{\left(E_{i}-E_{0}\right)}{E_{0}}\times 100\% \end{array}$$

Where, the variable *R* is the rate of inhibition or stimulation (negative for inhibition), expressed as a percentage. *E*_*i*_ and *E*_0_ correspond to the test sample and control sample (without Cd added), respectively.

Hormetic dose—response relationship is commonly assessed quantitatively by estimating the magnitude of the stimulatory response and the concentration range (width) of the hormetic zone. Derived from fitting curves, *M*_*max*_ (maximum stimulation rate) is utilized. Furthermore, *Hor*_*zone*_ (stimulation area) is employed to integrate stimulation amplitude and maximum stimulation rate into a unified parameter that effectively describes hormetic features in organisms (Supplementary Figure S1).

### 2.4 Statistics and analysis

All treatments were repeated three times, and one-way analysis of variance (ANOVA) and Duncan's new multiple range test were used to compare the differences in bacterial relative abundance, diversity, and DOM concentration among different heterogeneity treatments. The tests were conducted at a significance level of *p* < 0.05 and identified with letters. Results were expressed as arithmetic mean ± standard deviation (mean ± SD). Statistical analysis was performed using IBM SPSS Statistics 26 (Armonk, New York, USA).

The LogNormal Model (peak type, 4 parameters) was employed to fit the dose-response curve using Sigmaplot 14.0 (Systat Software, Inc., California, USA). Additionally, OriginPro 2021 (OriginLab, Massachusetts, USA) was utilized for integration and calculation of the area under the inverted U-shaped curve (*Hor*_*zone*_). All the graphs were plotted using Origin 2021.

## 3 Result

### 3.1 Soil respiration rate

**Figure 2:**
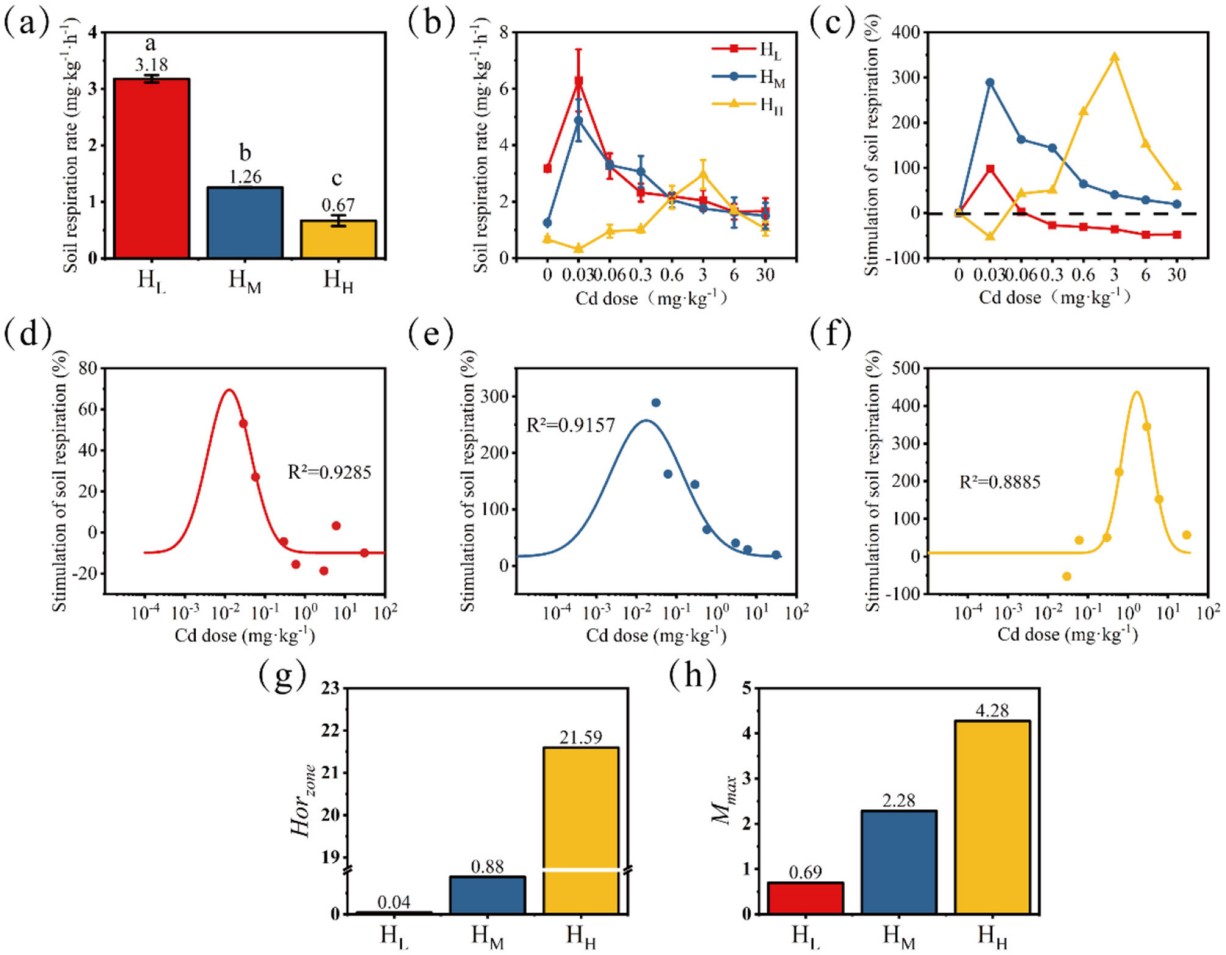
Changes in soil respiration rate without Cd stress **(a)** and with Cd stress **(b)**; the trend of stimulation rate was calculated with the no-stress treatment as the baseline **(c)**. Differences among means were considered statistically significant at *p* < 0.05, which were labeled with different lowercase letters. Dose-response relationships fitted based on the stimulation rate (**d**, H_L_; **e**, H_M_; **f**, H_H_) and characteristic values of the fitted curves *Hor*_*zone*_ **(g)** and *M*_*max*_ **(h)** were presented.

After 24 h of cultivation, in the treatments without Cd stress, the respiratory rate gradually decreased from low to high according to the degree of heterogeneity (Figure 2a). The treatment with the lowest heterogeneity (H_L_) had a respiratory rate of 3.18 mg·kg^−1^·h^−1^, while the treatment with the highest heterogeneity (H_H_) had a respiratory rate of 0.67 mg·kg^−1^·h^−1^. This indicates that the treatment with a higher degree of nutrient heterogeneity lost less C through soil respiration.

After 24 h of Cd exposure, the changes in soil respiration rate are shown in Figure 2b, and the stimulation rate (sti, %) calculated based on the control (without stress) is shown in Figure 2c. When the Cd dose was 0.03 mg·kg^−1^, the stimulation amplitude of soil respiration rate in the H_L_ treatment was the largest, reaching 98.1%, and the respiration rate was 6.3 mg·kg^−1^·h^−1^ at this time. When the Cd stress dose was 0.03–0.3 mg·kg^−1^, the soil respiration rate in the H_M_ treatment was significantly higher than that in the control, with stimulation rates ranging from 144.0 to 288.9%. When the Cd stress dose was 0.6–6 mg·kg^−1^, the soil respiration rate in the H_H_ treatment was significantly higher than that in the control, with stimulation rates ranging from 152.0 to 344.5%. It is worth noting that before the appearance of the stimulation effect (i.e., at the Cd dose of 0.03 mg·kg^−1^), a significant inhibition occurred, with an inhibition rate of 53.2%. At the same time, as the heterogeneity degree increased, the compensatory stimulation after stress became higher.

Based on the results of the stimulation rate, the data were fitted and calculated to describe the differences in the Hormesis characteristics of different treatments (Figures 2d–f). The order of *Hor*_*zone*_ from large to small is: H_H_ > H_M_ > H_L_ (Figure 2g). The order of *M*_*max*_ from large to small is: H_H_ > H_M_ > H_L_ (Figure 2h). Both *M*_*max*_ and *Hor*_*zone*_ increase with the enhancement of nutrient heterogeneity. This further indicates that the treatment with a high degree of nutrient heterogeneity is more “resilient” in the face of stress, as shown by a higher stimulation rate and a wider stimulation dose.

### 3.2 Microbial communities

#### 3.2.1 Relative abundance at phylum level

**Figure 3:**
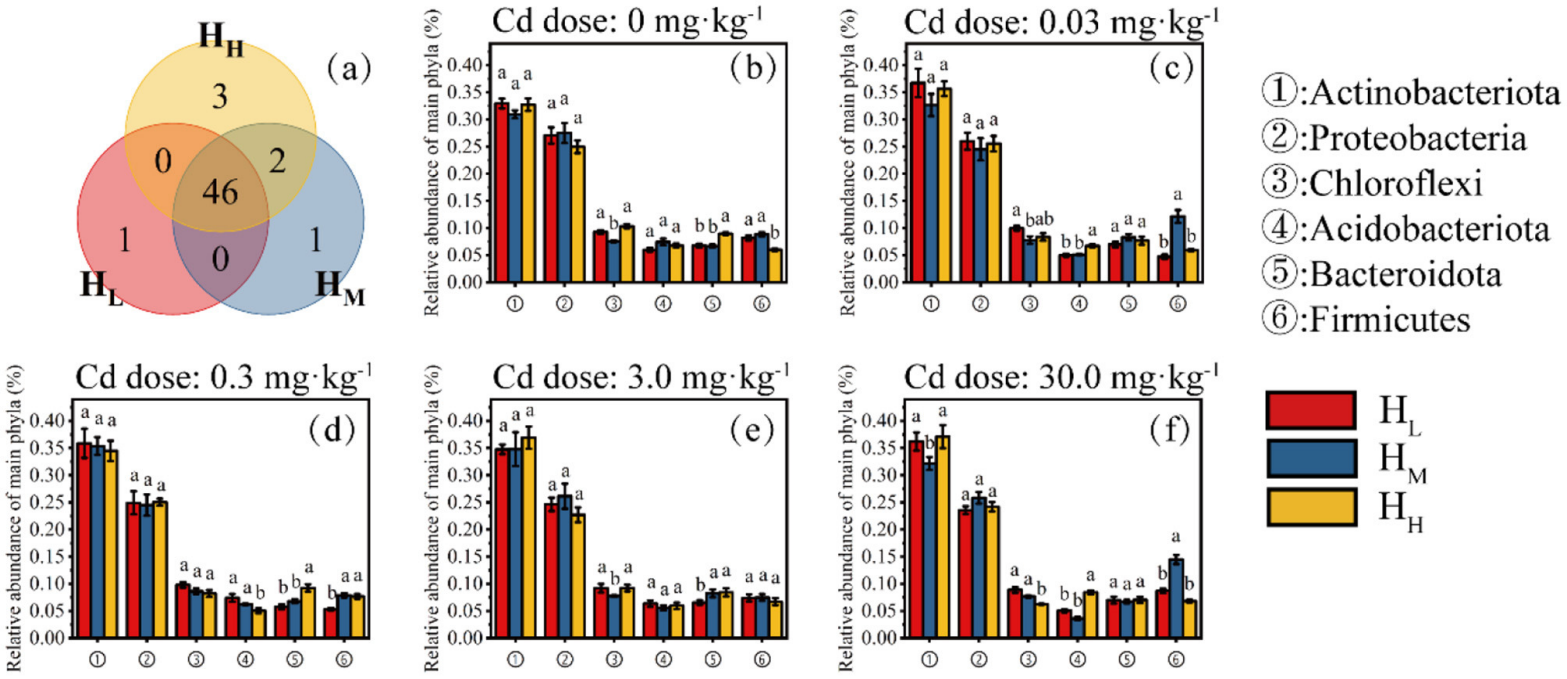
Distribution characteristics of phyla (Venn, **a**) and relative abundance of six major phyla at different Cd doses (**b**, 0; **c**, 0.03 mg·kg^−1^; **d**, 0.3 mg·kg^−1^; **e**, 3.0 mg·kg^−1^; **f**, 30 mg·kg^−1^).

At the phylum level, a total of 53 bacterial phyla were detected in the H_L_, H_M_, and H_H_ treatments, with 47, 46, and 50 phyla detected, respectively. There is one phylum that only appears in the H_L_ treatment, one in the H_M_ treatment, and three in the H_H_ treatment (Figure 3a).

The six main bacterial phyla of concern were Actinobacteriota, Proteobacteria, Chloroflexi, Acidobacteriota, Bacteroidota, and Firmicutes. The phylum Actinobacteriota, which had the highest relative abundance, showed significant differences among the different heterogeneity treatments only at a Cd concentration of 30 mg·kg^−1^, with the lowest relative abundance in the H_M_ treatment at 32.1% (Figure 3f). The relative abundances of the Proteobacteria, Chloroflexi, Acidobacteriota, and Bacteroidota did not show significant differences among all doses and heterogeneity treatments. Notably, the relative abundance of the Firmicutes was higher in the H_M_ treatment than in the H_L_ and H_H_ treatments under all Cd doses, and was significantly higher than the other two treatments at Cd doses of 0.03 and 30 mg·kg^−1^ (Figures 3b–e).

#### 3.2.2 Relative abundance at genus level

**Figure 4:**
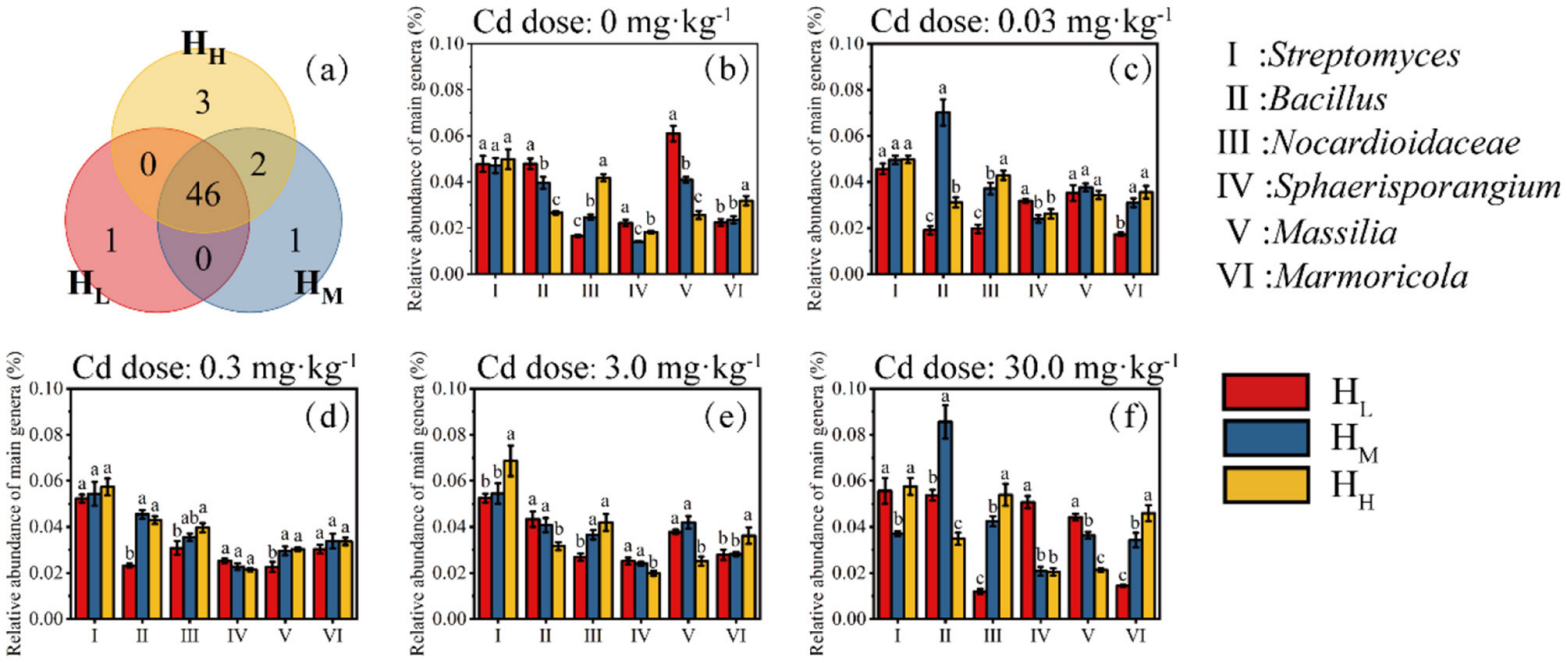
Distribution characteristics of genera (Venn, **a**) and relative abundance of six major genera at different Cd doses (**b**, 0; **c**, 0.03 mg·kg^−1^; **d**, 0.3 mg·kg^−1^; **e**, 3.0 mg·kg^−1^; **f**, 30 mg·kg^−1^).

At the genus level, a total of 1,239 bacterial genera were detected in the H_L_, H_M_ and H_H_ treatments, with 1,013, 1,106, and 1,120 genera detected, respectively. There are 25 genera that only appears in the H_L_ treatment, 67 in the H_M_ treatment, and 72 in the H_H_ treatment (Figure 4a).

The relative abundance of the *Streptomyces* did not show significant differences when the Cd dose was from 0 to 0.3 mg·kg^−1^ (Figures 4b–d). However, when the Cd dose was 3.0 mg·kg^−1^, the relative abundance in the H_H_ treatment was significantly higher than that in the H_L_ and H_M_ treatments (Figure 4e). When the Cd dose was 30.0 mg·kg^−1^, the relative abundance in the H_M_ treatment was significantly lower than that in the H_L_ and H_H_ treatments. The relative abundance of the *Bacillus* in the H_M_ treatment was significantly higher than that in the H_L_ and H_H_ treatments when the Cd dose was 0.03 and 30 mg·kg^−1^ (Figure 4f). The relative abundance of the *Nocardioidaceae* increased significantly with the increase of heterogeneity in all Cd treatments. The relative abundance of the *Marmoricola* also showed a similar phenomenon to that of the *Nocardioidaceae*.

#### 3.2.3 α-diversity

**Figure 5:**
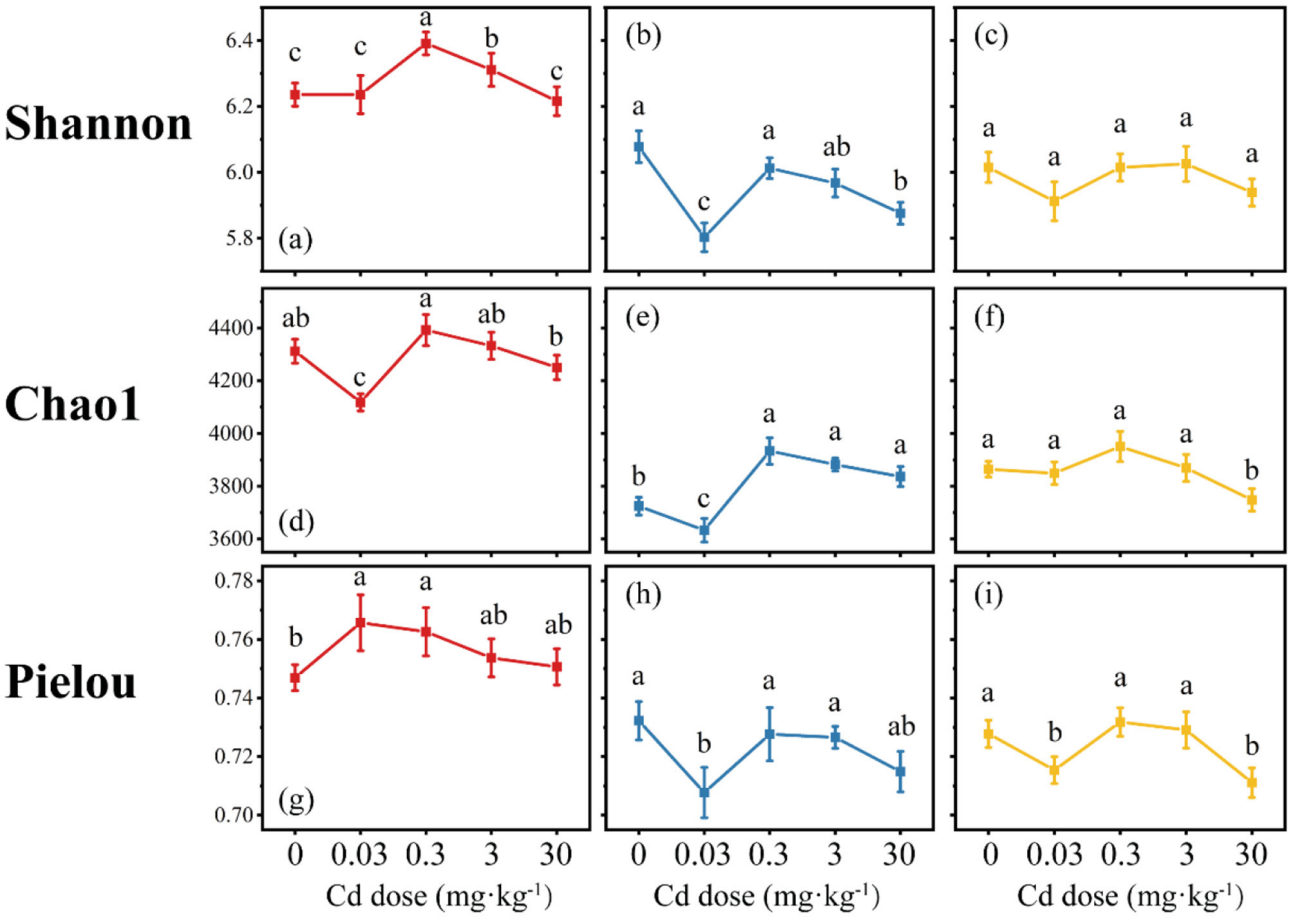
Changes in soil microbial α-diversity index (red, H_L_; blue, H_M_; yellow, H_H_). Differences among means were considered statistically significant at p < 0.05, which were labeled with different lowercase letters (**a–c**, Shannon Index; **d, e**, Chao1; **g–i**, Pielou Index).

The Shannon diversity index of the H_L_ treatment was the highest and significantly higher than that of the H_M_ and H_H_ treatments, with an average value of 6.3 (Figures 5a–c). The Chao1 index showed that the Chao1 index of the H_L_ treatment was significantly higher than that of the H_M_ and H_H_ treatments, with an average value of 4280.9 (Figures 5d–f). The distribution range of the Chao1 index in the H_M_ treatment under Cd stress was smaller than that in the H_H_ treatment, and the average richness of the H_M_ treatment was lower than that of the H_H_ treatment, being 3801.7 and 3856.2, respectively. The average Pielou index of the H_L_ treatment was higher than that of the H_M_ and H_H_ treatments, but not significantly. The average values of the three treatments were 0.76, 0.72, and 0.73, respectively. The impact of Cd stress on the distribution range of the Pielou index was the greatest in the H_M_ treatment, ranging from 0.71 to 0.74 (Figure 5g), followed by the H_H_ treatment, ranging from 0.71 to 0.73 (Figure 5h), and the smallest in the H_L_ treatment, ranging from 0.75 to 0.77 (Figure 5i).

Overall, regardless of whether under Cd stress or not, the soil bacterial diversity, richness, and evenness in the H_L_ treatment were higher than those in the H_M_ and H_H_ treatments. Meanwhile, the impact of Cd stress on the soil bacterial diversity, richness, and evenness in the H_H_ treatment was the smallest. This indicates that as the degree of nutrient heterogeneity increases, the soil bacterial community is further screened, the abundance of dominant species becomes more concentrated, and the ability to cope with stress also becomes stronger.

### 3.3 DOM

**Figure 6:**
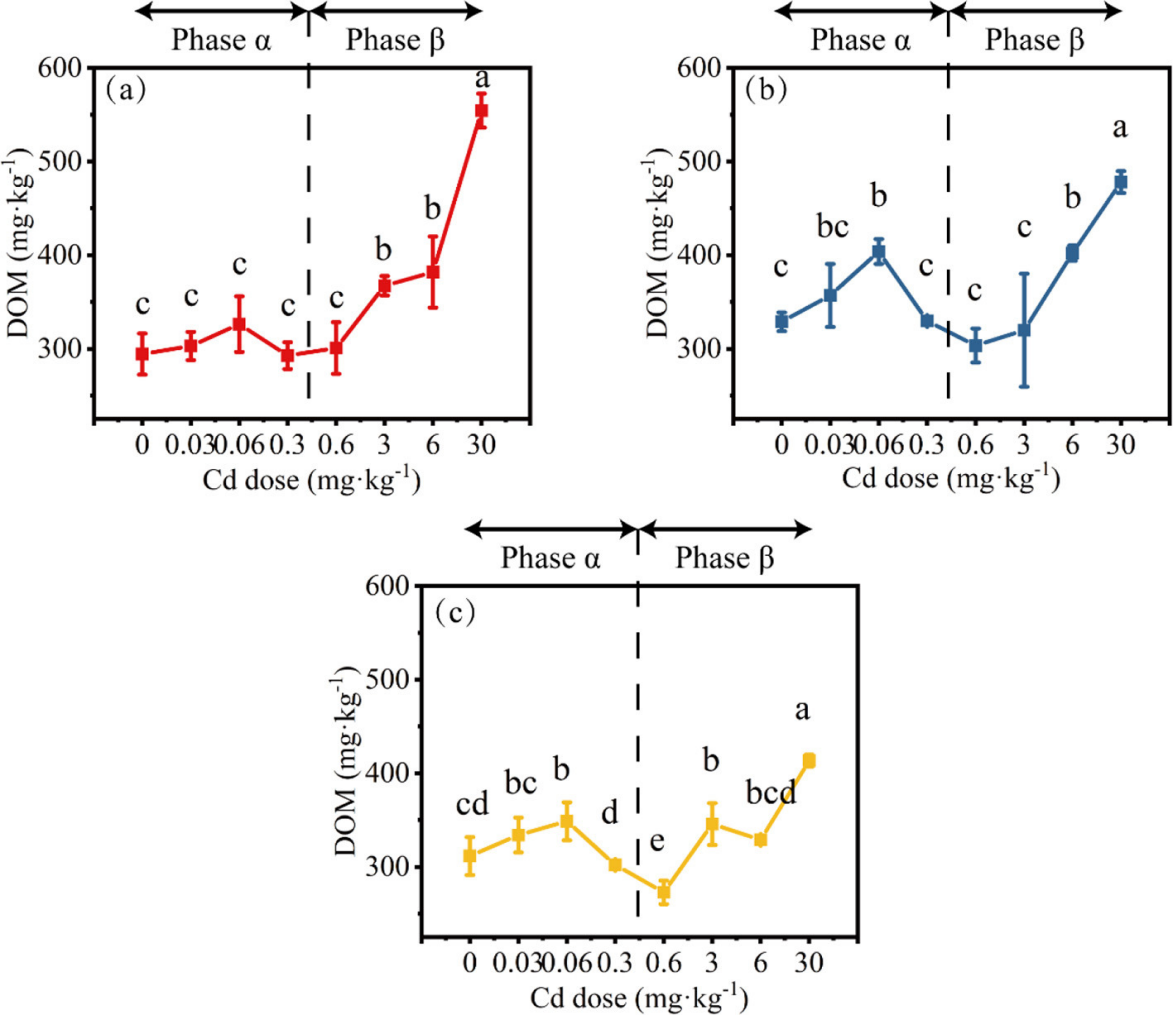
The changes in DOM concentration under different conditions (**a**, H_L_; **b**, H_M_; **c**, H_H_). Differences among means were considered statistically significant at *p* < 0.05, which were labeled with different lowercase letters. The Phase α and β are separated by 0.6 mg·kg^−1^ Cd (0.6 mg·kg^−1^ Cd belongs to the Phase β).

In the H_L_ treatment, when the Cd dose was 0–0.6 mg·kg^−1^, the DOM content did not show significant changes (the peak value within 0–0.6 mg·kg^−1^ Cd increased by 10.8% compared to 0 Cd). However, when the Cd dose exceeded 0.6 mg·kg^−1^, the DOM content rapidly increased, reaching 554.3 mg·kg^−1^ (Figure 6a). In the H_M_ treatment, the peak DOM content at 0.06 mg·kg^−1^ Cd was significantly higher than that at 0.3–3 mg·kg^−1^ Cd, reaching 403.9 mg·kg^−1^ (increased by 22.8% compared with 0 Cd; Figure 6b). The peak DOM content in the H_H_ treatment also occurred at 0.06 mg·kg^−1^ Cd, reaching 348.7 mg·kg^−1^ (increased by 11.9% compared with 0 Cd; Figure 6c). When the Cd dose was >3 mg·kg^−1^, the DOM content in both H_M_ and H_H_ treatments increased, and this change was consistent across all treatments.

The 3D fluorescence spectra of all samples were collected by fluorescence excitation-emission matrix spectra (EEMS), and the typical spectra are shown in Supplementary Figures S2b, c. The fluorescence information was decomposed into four main components with the use of parallel factor analysis (Supplementary Figures S2a, S3). The samples did not show significant differences or Hormesis-like phenomenon (Supplementary Figure S4).

Overall, when the Cd dose was < 0.6 mg·kg^−1^ (Phase α), under conditions of lower heterogeneity (H_L_ treatment), the DOM content tended to increase with the increase in Cd dose; while under conditions of higher heterogeneity (H_M_ and H_H_ treatments), a typical hormesis phenomenon was observed. When the Cd dose was >0.6 mg·kg^−1^ (Phase β), regardless of the heterogeneity treatment, the DOM content increased with the increase in Cd dose. No significant effect on DOM content under 0 Cd was observed regardless of the type of heterogeneity treatment.

### 3.4 Dose-synchronous relationship between soil respiration and microbial communities

**Figure 7:**
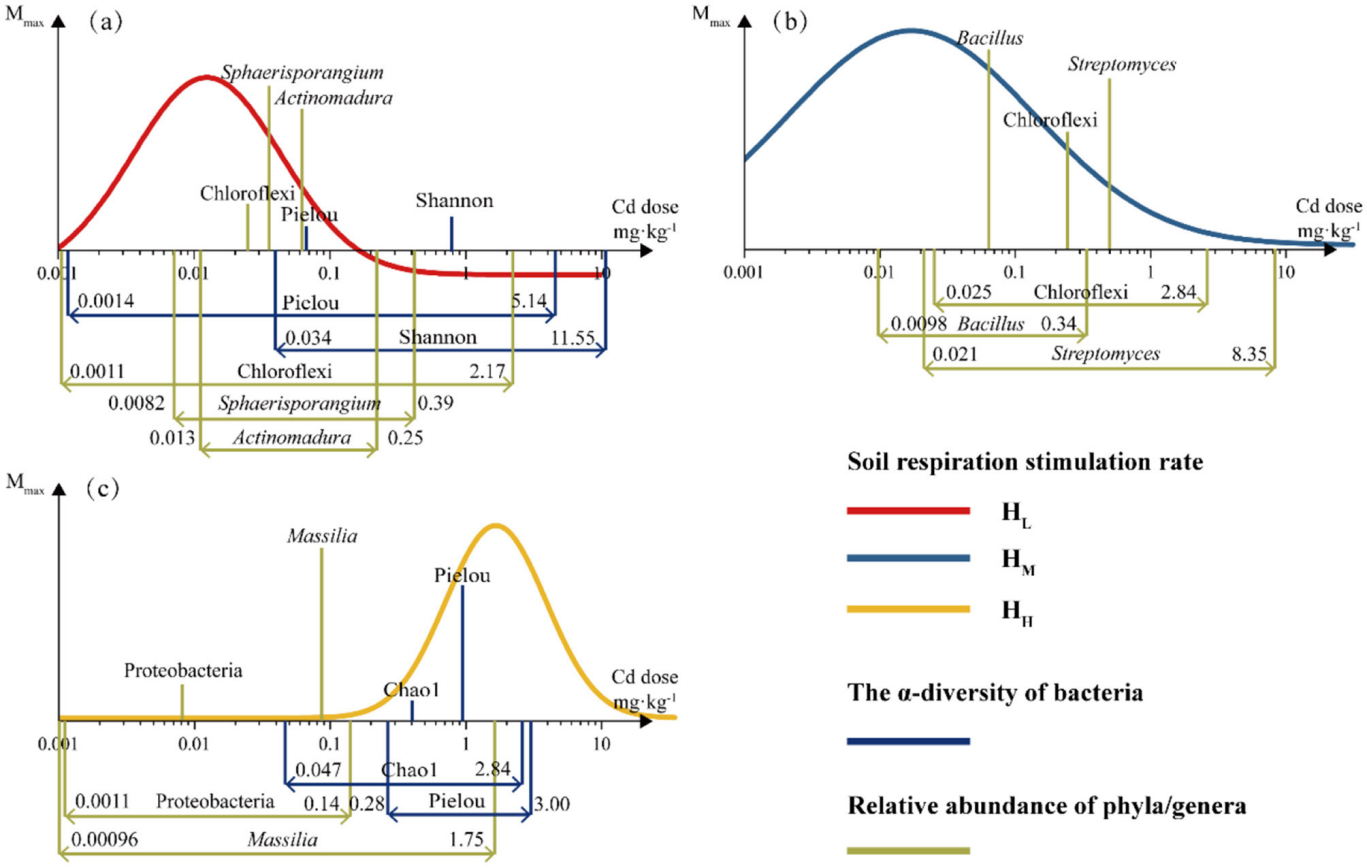
The dose-synchronous responses of soil respiration rate and microbial relative abundance and diversity to Cd stress. The significance of all the fits was < 0.05 and *R*^2^ was shown in Supplementary Table S3. The height of the column represents the Mmax and the range marked at the bottom indicates the range of response doses (**a**, H_L_; **b**, H_M_; **c**, H_H_).

The data of all endpoints at each dose were converted into stimulation rates and fitted. Based on the fitting results, the parameters with hormesis-like characteristics in the dominant bacterial phyla and genera and α-diversity were fitted and summarized in the Figure 7. The figure shows the hormesis fitting curve of soil respiration rate and the *M*_*max*_ and hormesis dose range of the endpoints with hormesis-like characteristics.

It can be observed that in the treatments with lower heterogeneity (H_L_, H_M_), the dose at which soil respiration rate reached *M*_*max*_ was lower than bacterial abundance and α-diversity, while this was not the case in H_H_. In terms of the range of stimulation doses, the response of soil respiration (0.2–16.3 mg·kg^−1^) is greater than that of α-diversity (0.3–18.3 mg·kg^−1^) and bacterial abundance (0.1–4.9 mg·kg^−1^) and almost covers the ranges of both. The endpoints with the greatest *M*_*max*_ were all microbial categories, namely *Sphaerisporangium, Bacillus*, and *Massilia*. This indicates that under different heterogeneous treatments, the main microbial groups that dominate the response to Cd stress and exhibit hormesis-like characteristics are different. The response of α-diversity lags behind that of microorganisms, and the responses of diversity (Shannon) and richness (Chao1) do not show a high *M*_*max*_. Although soil respiration is an indication of the overall metabolic status of soil microorganisms, the lagging and low Mmax response of α-diversity are not contradictory to the high response characteristics of specific microorganisms (Supplementary Table S3).

## 4 Discussion

The influence of soil heterogeneity on global change (GC) has been studied for a long time, and most of these influences are mediated by plants, soil animals and microorganisms (Bardgett et al., 2013). The most intuitive effect of plant-mediated soil heterogeneity is the stimulation of primary productivity (Hodge, 2004; Kembel and Cahill, 2011; Richardson et al., 2009). In non-plant-mediated soil heterogeneity-related studies, the bioturbation of soil animals (such as the burrowing activities and excrement enrichment) and the utilization of existing nutrients by microbial communities are regarded as the main causes of soil heterogeneity (Lavelle et al., 2020). Therefore, the competition among microorganisms for nutrients cannot be fully understood without explicitly considering soil heterogeneity. At the same time, it has been reported that the mutualistic interaction between plant roots and fungi is a case of homogenization promoting productivity increase [+31% net primary production (Van Der Heijden et al., 2008)]. It can be seen that the homogenization or heterogeneity of soil plays a decisive role in global climate change and local nutrient cycling (Schimel and Bennett, 2004). Given that the competitive relationship of microorganisms for nutrients is affected by the increase in atmospheric CO_2_ concentration or climate change, we believe that soil heterogeneity plays a regulatory role in the changes of GC.

Our research indicates that the soil respiration rate in landscapes with a low degree of heterogeneity is higher than that in landscapes with a high degree of heterogeneity. This implies that the C loss caused by soil respiration is smaller in landscapes with a high degree of heterogeneity. Currently, there is no clear consensus on the reports regarding the influence of nutrient heterogeneity on soil respiration. Some indoor simulation experiments can support our conclusion (Xiao et al., 2018; Xue et al., 2016, 2021; Shen et al., 2019), while some outdoor experiments have differences (Jing et al., 2020; Nunan et al., 2020; Gonod et al., 2006; Dong et al., 2015). The reasons might be the neglect of soil texture (the ratio of clay, silt, and sand particles), soil moisture content (in the vertical or horizontal direction), and the heterogeneity of nutrient availability. Among them, nutrient availability is directly related to the soil respiration rate (Nwachukwu and Pulford, 2011; Niklińska et al., 2006). The reduction in respiration means that, compared to homogeneous soil landscapes, in landscapes with a high degree of heterogeneity, the respiration of some microorganisms is inhibited. We speculate that in landscapes with a high degree of heterogeneity, microorganisms in both high and low nutrient patches are subject to similar screening stress.

The results of high-throughput sequencing support this inference of ours. Among the main phylum of the community, three did not show significant differences (Actinobacteriota, Proteobacteria, Acidobacteriota) between the heterogeneous treatment and the control (*p* < 0.05). Among the main genus, there were many groups that showed a stepped distribution with the increase of heterogeneity degree. Among them, *Nocardioidaceae* and *Marmoricola* were positively correlated, and *Bacillus* and *Massila* were negatively correlated, while *Streptomyces*, with the highest relative abundance, did not show significant differences. This indicates that the community at the genus level is subject to the screening stress of nutrient heterogeneity, while this stress is not significant at the phylum level. This difference in classification hierarchy is in line with the “functional redundancy hypothesis” of stress adaptation (Fierer et al., 2012), that is, the conservative traits at the phylum level maintain stability, while the recombination of functional genes at the genus level leads to changes in respiratory metabolism (Zhou et al., 2014). There are also studies suggesting that this is an adaptive performance after being stressed, and the inhibition of soil respiration is one of the manifestations of the overall functional transformation of the community (Zalaghi et al., 2019).

When the community under the screening stress of heterogeneity is exposed to a new stress factor (Cd), it shows a completely different trend of respiratory rate change compared to the homogeneous state. In terms of the change in the stimulation rate (*M*_*max*_), the treatment with a high degree of heterogeneity is greater than the control. This indicates that heterogeneity enhances the “resilience” of the landscape ecosystem, that is, when subjected to the same stress, it exhibits more compensatory behaviors [Including higher soil respiration compensation and delayed soil respiration compensation (Scheffer et al., 2001)]. Some studies have described this compensatory behavior and summarized it as the “1/10 principle” (Calabrese, 2015). The results of *Hor*_*zone*_ also support our conclusion. In terms of the triggering dose of stimulation (*x*_0_), it gradually increases with the increase in the degree of heterogeneity. This indicates that the highly heterogeneous landscape has a stronger buffering capacity against stress. Systems with weak buffering capacity will compensate earlier, and the compensation intensity is lower than the systems with later compensating. Regarding buffering capacity and compensation intensity, they are two sides of the same coin in community stability. Buffering capacity is the ability of the system to maintain a steady state, while the compensation intensity is the ability to construct a new steady state. These two points reflect the two mechanisms of Hormesis, namely the “Overcompensation” and the “Overcorrection” (Tang et al., 2025; Calabrese et al., 2008; Sebastiano et al., 2022; Agathokleous et al., 2021). Although our experimental phenomena cannot clearly support one of them, both can explain the phenomenon of the simultaneous backward shift of the stimulation rate and the stimulation dose in the experiment.

The changes in DOM present an interesting trend, which is divided into the α and β phases with 0.6 mg·kg^−1^ as the boundary. The α phase shows a hormesis-like phenomenon, while the β phase shows a monotonous increase. This is a common phenomenon in all treatments, and it is speculated to be caused by the difference in nutrient heterogeneity set. In the introduction, we assumed that the level of nutrient content and the length of the corridors formed by adjacent patches will changes the composition and interrelationships of microbial community. Although the specific process of this change is still unclear, it is certain that this change exists objectively and is reflected in the change of DOM content. The high-dose Cd stress masks the difference brought by nutrient heterogeneity, while the Hormesis-like phenomenon at the low dose is closely related Quorum sensing (QS) of microorganism. There are researches finding that nutrient heterogeneity promotes the transformation of small molecule DOM by changing QS signal molecules [such as Acyl Homoserine Lactone (AHLs); Zhang et al., 2022]. Another study found that the death of some microorganisms caused by high concentrations of Cd will increase the DOM content (Zhang et al., 2009). Therefore, a possible explanation for the Hormesis-like phenomenon in the low-dose range is that the microbial community converts more macromolecular insoluble organic matter into small molecule soluble organic carbon in order to adapt to the environment, and the nutrient heterogeneity further strengthens the transformation effect of this ecological function.

In conclusion, if heterogeneity is understood as a disturbance that alters the steady state of the current ecosystem, and the changes brought about by the disturbance are regarded as QS of the communities within the system. Assuming that the system has a corresponding compensation for each stress, then when an ecosystem under stress is subjected to a new stress, its compensation will be greater than that of the system not under stress. This inference unifies the stresses imposed on the system at different levels and uses the intensity of the compensatory effect of the community in the face of new stresses (i.e., the standard Hormesis experimental model) to describe the current stability and resilience of the community. We believe that it is necessary to further study the quantitative methods in order to promote a new method that can comprehensively describe the quality of the soil ecosystem.

## 5 Conclusion

We conclude that Soil respiration is regulated by Cd and exhibits an inverted U-shaped dose-response curve. The spatial distribution of nutrients delays the occurrence of maximum respiration rate in response to stress and increases *Hor*_*zone*_. In terms of changes in DOM content, high doses of Cd stress (>0.6 mg·kg^−1^) mask the differences in the effects of spatial heterogeneity, while at low Cd doses ( ≤ 0.6 mg·kg^−1^), there may be a possible occurrence of hormesis-like phenomenon closely related to DOM stability and microbial response under spatial heterogeneity treatment. Chloroflexi and Proteobacteria are major phyla with significant compensatory effects on Cd stress under nutrient spatial heterogeneity treatment. The influence pathway of nutrient spatial heterogeneity on Cd-induced soil respiration hormesis shows that the relative abundance of sensitive bacteria responded first (0.1–4.9 mg·kg^−1^), followed by the α-diversity (0.3–18.3 mg·kg^−1^), while the DOM content did not respond significantly. Soil respiration compensation effect (0.2–16.3 mg·kg^−1^) almost synchronizes with α-diversity. We verified the impact of cadmium stress on the nutrient spatial heterogeneity mediated by DOM and microorganisms in soil respiration and demonstrated hormone effects. Additionally, we proved that bacterial community changes play a crucial role in this process.

## Data Availability

The original contributions presented in the study are publicly available. This data can be found here: https://www.ncbi.nlm.nih.gov/, accession number: PRJNA1299094.
