# Supplementary material for: New perspectives under heterogeneity: mechanism of nutrient heterogeneity on Cd-induced hormesis of soil respiration
Source: Front Microbiol. 2025 Aug 12;16:1647658. doi: 10.3389/fmicb.2025.1647658 (PMC12379143; doi:10.3389/fmicb.2025.1647658)
Supplement: Supplementary file 1 [file Supplementary_file_1.docx]

# **New perspectives under heterogeneity: mechanism of nutrient heterogeneity on Cd-induced Hormesis of soil respiration**

## Tables

**Table S1** Physical and chemical properties of tested soil (mean ± SD, n=5)

| Soil type | pH | EC  (mS·cm^-1^) | SOC  (g·kg^-1^) | TN  (g·kg^-1^) | TP  (g·kg^-1^) |
| --- | --- | --- | --- | --- | --- |
| forest soil | 7.72±0.01 | 0.91±0.06 | 17.22±1.21 | 1.33±0.15 | 0.56±0.04 |
| organic medium | 6.42±0.15 | 4.01±0.10 | 421.12±50.62 | 42.23±12.48 | 38.54±8.87 |

**Table S2** Weight of the tested soil forming the patch type

| Patch type | Forest soil  (g) | Organic medium  (g) |
| --- | --- | --- |
| N_L_ | 800 | 0 |
| N_M_ | 600 | 80 |
| N_H_ | 400 | 160 |

**Table S3** Fitting results of homesis parameters

| Patch type | Test endpoints | *x_1_* | *x_2_* | *Q_i_* | *Hor_zone_* | *M_max_* | R^2^ |
| --- | --- | --- | --- | --- | --- | --- | --- |
| H_L_ | Shannon | 0.04 | 11.55 | 339.83 | 0.09 | 0.04 | 0.99 |
|  | Pielou_e | ＜0.01 | 5.14 | 3651.75 | 0.05 | 0.03 | 0.94 |
|  | Chloroflexi | ＜0.01 | 2.17 | 2016.92 | 0.06 | 0.08 | 0.99 |
|  | *Actinomadura* | ＜0.01 | 0.40 | 47.83 | 0.11 | 0.57 | 0.92 |
|  | *Sphaerisporangium* | 0.02 | 0.26 | 19.46 | 0.08 | 0.65 | 0.87 |
| H_M_ | Chloroflexi | 0.03 | 2.84 | 115.48 | 0.18 | 0.14 | 0.99 |
|  | *Bacillus* | ＜0.01 | 0.35 | 34.92 | 0.17 | 1.06 | 0.99 |
|  | *Streptomyces* | 0.02 | 8.36 | 399.55 | 0.62 | 0.20 | 0.69 |
| H_H_ | Chao1 | 0.05 | 2.84 | 60.43 | 0.04 | 0.03 | 0.67 |
|  | Pielou_e | 0.28 | 3.00 | 10.75 | 0.19 | 0.15 | 0.56 |
|  | Proteobacteria | ＜0.01 | 0.14 | 121.89 | 0.01 | 0.04 | 0.66 |
|  | *Massilia* | ＜0.01 | 1.75 | 1810.56 | 0.19 | 0.33 | 0.96 |

**Table S4** Soil microbial α-diversity index (mean ± standard deviation)

| α-diversity index | Cd dose  (mg·kg^-1^) | HL  (mean±sd) | | HM  (mean±sd) | | HH  (mean±sd) | |
| --- | --- | --- | --- | --- | --- | --- | --- |
| Shannon | 0 | 6.24 | 0.04 | 6.08 | 0.05 | 6.02 | 0.05 |
|  | 0.03 | 6.24 | 0.06 | 5.80 | 0.04 | 5.91 | 0.06 |
|  | 0.3 | 6.39 | 0.03 | 6.01 | 0.03 | 6.02 | 0.04 |
|  | 3 | 6.31 | 0.05 | 5.97 | 0.04 | 6.03 | 0.05 |
|  | 30 | 6.22 | 0.04 | 5.88 | 0.03 | 5.94 | 0.04 |
| Chao1 | 0 | 4311.89 | 45.03 | 3724.21 | 33.60 | 3864.37 | 29.86 |
|  | 0.03 | 4118.08 | 32.34 | 3632.82 | 44.91 | 3849.19 | 42.79 |
|  | 0.3 | 4392.05 | 59.20 | 3933.50 | 50.31 | 3950.28 | 57.42 |
|  | 3 | 4332.40 | 51.51 | 3881.80 | 24.24 | 3869.17 | 50.89 |
|  | 30 | 4250.27 | 46.40 | 3836.37 | 38.00 | 3747.83 | 42.85 |
| Pielou | 0 | 0.75 | 0.00 | 0.73 | 0.01 | 0.73 | 0.00 |
|  | 0.03 | 0.77 | 0.01 | 0.71 | 0.01 | 0.72 | 0.00 |
|  | 0.3 | 0.76 | 0.01 | 0.73 | 0.01 | 0.73 | 0.00 |
|  | 3 | 0.75 | 0.01 | 0.73 | 0.00 | 0.73 | 0.01 |
|  | 30 | 0.75 | 0.01 | 0.71 | 0.01 | 0.71 | 0.01 |

**Table S5** Pearson correlation between soil respiration and DOM content

| Pearson's r | H_L_ | H_M_ | H_H_ |
| --- | --- | --- | --- |
| r | -0.45 | -0.10 | -0.14 |
| p | 0.27 | 0.82 | 0.74 |

Note: The dataset conforms to a normal distribution.

## Figure


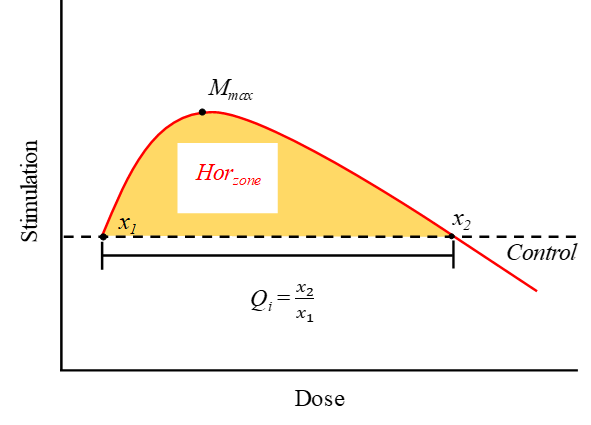


**Fig. S1** Model fitting and parameter setting for a quantitative evaluation of hormesis based on Fan et al. (2021)[1]. *M_max_* represents the maximum stimulatory magnitude; *Q_i_* means the stimulatory domain; *x_1_* and *x_2_* represent the zero-equivalent points at the beginning and end of the stimulus, respectively; *Hor_zone_* is hormetic zone, the stimulatory area of the U-shape curve (the yellow area).


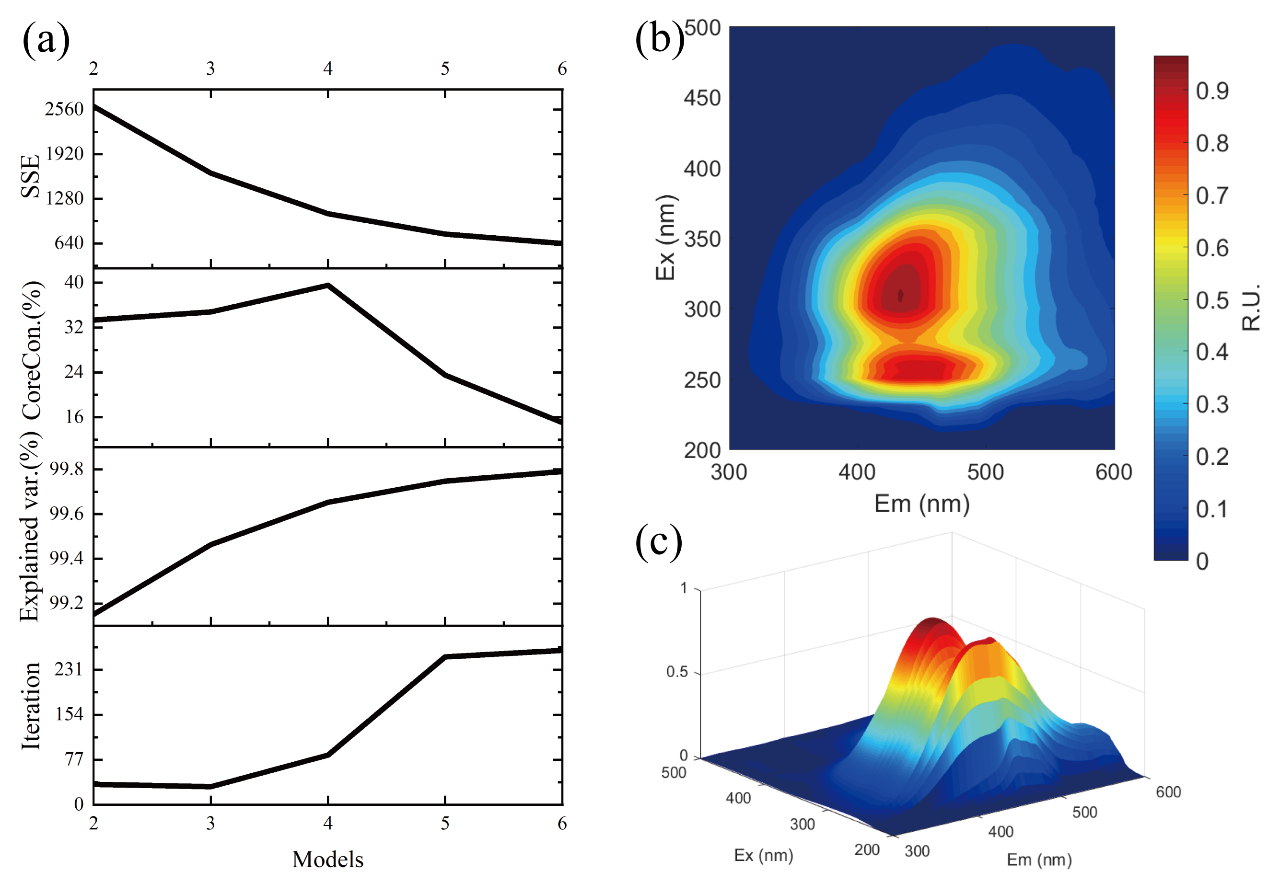


**Fig. S2** Representative 3D fluorescence spectra (b, 2D; c, 3D) and four parameters to determine the number of principal components by parallel factor analysis(a). (SSE, sum of squared errors; CoreCon, core consistency; Explained var, percentage of explanatory variables; Iteration, number of iterations)


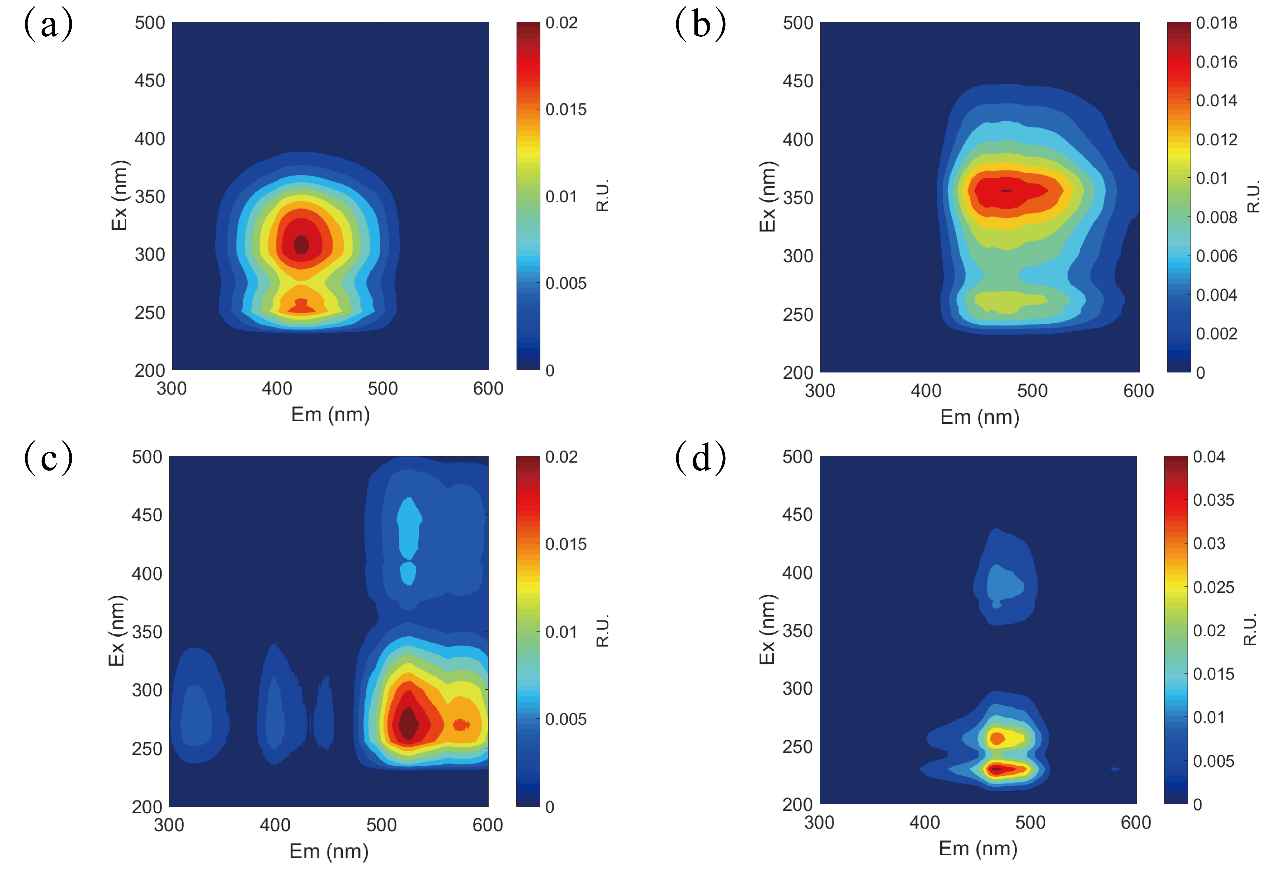


**Fig. S3** Four fluorescent components of soil DOM identified according to PARAFAC (a, C1; b, C2; c, C3; d, C4)


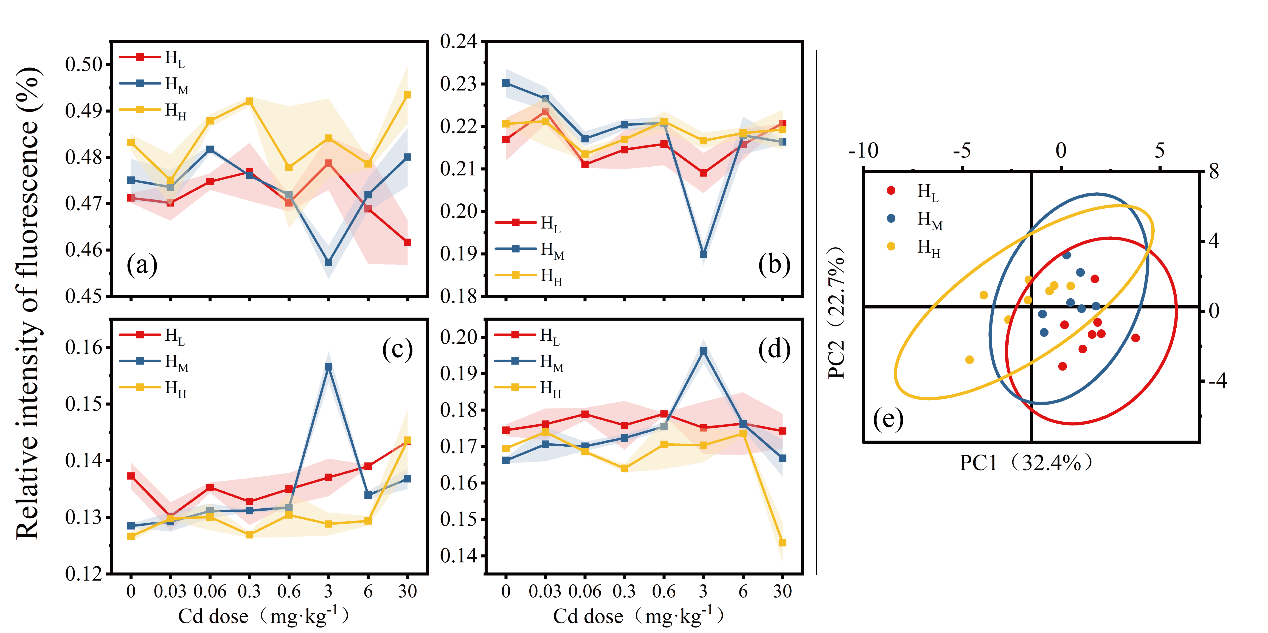


**Fig. S4** The relative fluorescence intensity changes of the four components with increasing Cd dose (mean ± SD, n=3; a, C1; b, C2; c, C3; d, C4), and principal component analysis (PCA) of all samples (e). The explanation rate of PCA reached 55.1%.


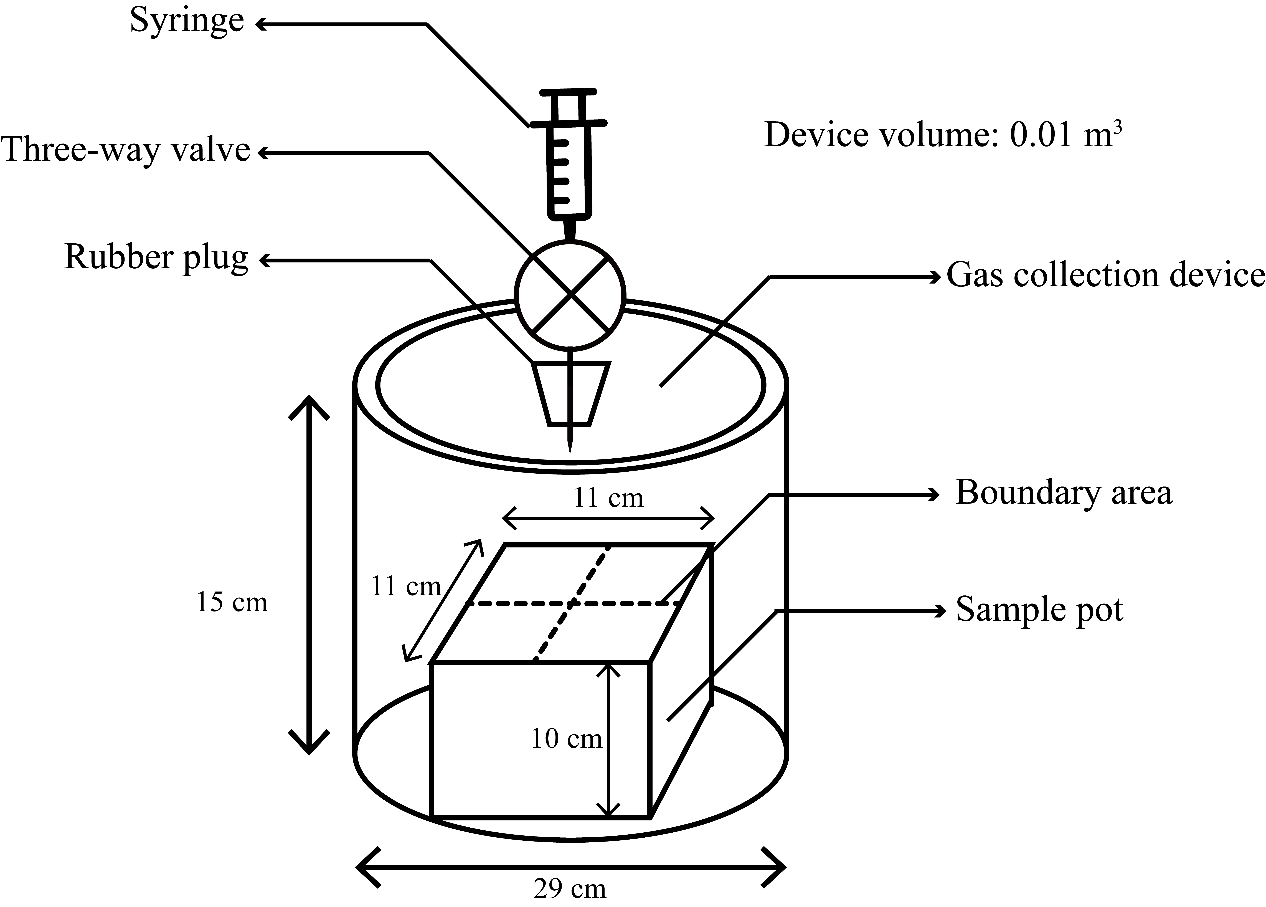


**Fig. S5** Schematic diagram of gas collection device (shielded from light during sampling)

## Citation

[1] D.W. Fan, J.W. Sun, C.L. Liu, S.Y. Wang, J.G. Han, E. Agathokleous, Y.L. Zhu, Measurement and modeling of hormesis in soil bacteria and fungi under single and combined treatments of Cd and Pb, Sci. Total Environ., 783 (2021) 147494.
